# Exploring cross-cultural variations in visual attention patterns inside and outside national borders using immersive virtual reality

**DOI:** 10.1038/s41598-023-46103-1

**Published:** 2023-11-01

**Authors:** Alžběta Šašinková, Jiří Čeněk, Pavel Ugwitz, Jie-Li Tsai, Ioannis Giannopoulos, David Lacko, Zdeněk Stachoň, Jan Fitz, Čeněk Šašinka

**Affiliations:** 1 Department of Information and Library Studies, Faculty of Arts, Masaryk University, Brno, Czech Republic; 2 Department of Social Studies, Faculty of Regional Development and International Studies, Mendel University in Brno, Brno, Czech Republic; 3 Department of Psychology, Research Center for Mind, Brain, and Learning, National Chengchi University, Taipei, Taiwan, ROC; 4 Department of Geodesy and Geoinformation, Vienna University of Technology, Vienna, Austria

**Keywords:** Psychology, Human behaviour

## Abstract

We examined theories of cross-cultural differences in cognitive style on a sample of 242 participants representing five cultural groups (Czechia, Ghana, eastern and western Turkey, and Taiwan). The experiment involved immersive virtual environments consisting of two salient focal objects and a complex background as stimuli, which were presented using virtual reality headsets with integrated eye-tracking devices. The oculomotor patterns confirmed previous general conclusions that Eastern cultures have a more holistic cognitive style, while Western cultures predominantly have an analytic cognitive style. The differences were particularly noticeable between Taiwan and the other samples. However, we found that the broader cultural background of each group was perhaps just as important as geographical location or national boundaries. For example, observed differences between Eastern (more holistic style) and Western Turkey (more analytic style), suggest the possible influence of varying historical and cultural characteristics on the cognitive processing of complex visual stimuli.

## Introduction

Past research in cross-cultural psychology suggests the existence of systematic differences in perceptual and cognitive domains across nations and cultural groups. This area of research covers a variety of specific cognitive processes, such as object categorization, thinking about contradictions, object-background separation, selective attention to objects and background in complex scenes, processing of global and local features of objects, attention to changes in the visual field, and many others^1^.

Following this area of research, a theory of holistic and analytic cognition has been developed^2^. The theory distinguishes two relatively stable modes of cognitive processing, referred to as holistic and analytic cognitive styles, and states that certain individuals should process information from the world prevalently in one of these two distinctive ways. According to this theory, holistic individuals (compared to their analytic counterparts) (a) use more intuitive and less rule-based strategies in object categorization^3, 4^, (b) use dialectical thinking instead of rules of formal logic, (c) are less effective in separating objects from the background^5^ (d) focus more on the background and the relationships between objects and less on the salient (focal) objects and their attributes^6^, (e) focus more on the global features than the local features of objects^7^, and (g) are more sensitive to contextual changes than focal object changes^8^.

Our current research focuses on one of these processes: selective attention to objects and their surroundings (background), sometimes also referred to as context sensitivity. Other authors have previously examined cross-cultural differences in context sensitivity using natural scenes (free-viewing paradigm) in combination with eye-movement recording^9–11^. Some research has found distinct differences in eye-movement patterns between Chinese and Americans^9^ and Chinese and Africans^10^, whereas other studies have supported a null hypothesis on the lack of any systematic cultural differences in people viewing scenes^11^.

In the present study, we sought to replicate the results of previous experiments on cultural differences in context sensitivity in complex visual environments and to extend this design by transferring the experimental procedure into immersive virtual reality with 3D compositions of virtual environments. This type of adaptation also has two potential benefits over the presentation of 2D scenes: (1) hypotheses on the third dimension of the visual world (depth) can be formulated, and (2) given the three-dimensional nature of the visual world, such research can be considered more ecologically valid for everyday visual experience. In this manner, we are responding to the challenge posed by Elvio Blini and his colleagues, who argued that “*depth is a fundamental, yet overlooked, dimension of human perception” and that “future studies in vision and perception should be depth aware*”^12^.

We created a series of static 3D scenes, each consisting of two focal objects placed in a semantically congruent background environment. Because the focal objects were designed to be visually salient, we hypothesized that they would be of relatively greater interest to analytic individuals. In accordance with i.a. Brouwer^13^, Cryns^14^, and Pontius^15^, we therefore expected that these participants would spend relatively more time gazing at the focal objects (focal object dwell time) than they would on the background and less salient objects located within the background, whereas holistic individuals would spend relatively less time on the focal objects.

To more closely investigate the possible differences in perception of depth, one of the focal objects was located nearer to the viewer in approximately half of the 3D scenes (for more information, see the “Methods” section). In these scenes, we hypothesized that the more distant focal object would likely blend more with the background for the analytic participants and that they would therefore dwell less on the distant focal object than the nearer focal object.

A relatively intricate part of the present study was determining a priori which individuals from which specific countries could be expected to exhibit predominantly holistic or analytic patterns in context sensitivity. Most of the research in the field has focused on comparisons between “Western” (USA, Europe) and “Eastern” (Japan, China) samples, with Western samples being relatively consistently more analytic than Eastern samples (for a review, see^1^). However, while this conceptualization might be understandable when comparing two culturally distant samples, it doesn't have to be necessarily understood as dichotomous when comparing multiple less distinct cultures.

Quite the contrary, the East–West dichotomy seems to be rather reductionist and potentially distorting. Moreover, it ignores other potentially influential factors such as characteristics of the environment, socioeconomic status, religion, or other individual-level factors. This is especially valid for large and culturally heterogeneous countries or countries that are not confined to North America or Asia. For example, Chatterjee^16^, demonstrates this in reference to India which is commonly perceived as “Eastern” and collectivistic. She argues that India is a very diverse country where some areas tend toward collectivism and others toward individualism. In order to reflect this approach we included two purposefully selected samples from Turkey, the geographically largest and the most populous of the countries included in this research (for details, see the “Research sample” section).

Furthermore, in line with suggestions made by Matsumoto and Yoo^17^, we explored the possible influence of individual-level variables such as the size of the municipality the participant lives in (measured by population), gender, age, experience with living in other countries, number of siblings, socioeconomic status, and study area to “unpack” their possible influence on dependent variables.

In summary, even though we reflect the limits of the original theoretical foundations within the analytic-holistic paradigm, we still find the above-mentioned cross-cultural theories of cognitive style as viable and able to detect differences in cognitive processes even at a more detailed level and with a wider spectrum of cultures.

**Figure 1:**
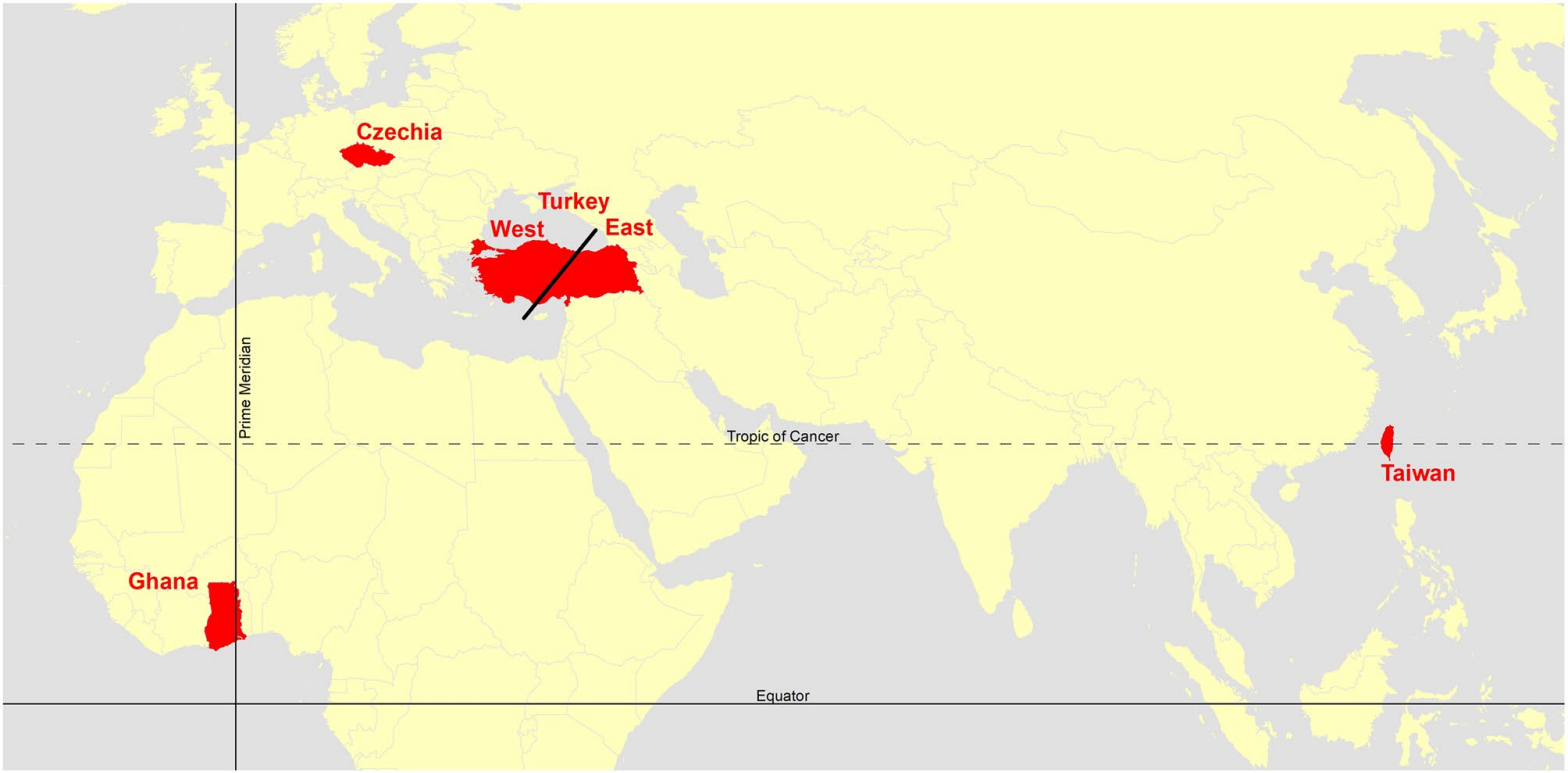
Map indicating the areas of origin of the participants*.* Created in ArcGIS Desktop 10.8, URL: https://www.esri.com/en-us/arcgis/products/arcgis-pro/overview.

Our research sample was composed of five cultural sub-samples from Czechia, Taiwan, Ghana, eastern Turkey, and western Turkey (Fig. 1). Based on previous research, we expected the Czech participants to be, on average, the most analytic of all the samples^6, 18^, whereas the Taiwanese, as East Asians, were the most holistic^9, 10^. The other three cultural samples should have fallen somewhere between these two samples in terms of context sensitivity. However, in the absence of any eye-tracking research that has applied complex scenes with a free-viewing paradigm to directly compare samples from the Middle East and Sub-Saharan Africa, we were unable to form any strong hypotheses about the holistic/analytic patterns of perception in these three samples.

When we included Turkey in the research, we were careful not to neglect the cultural variance within the country’s borders; much data on the socioeconomic development index (data from the Ministry of Development in^19^), fertility rate^20^ and ethnic distribution^21^ according to province indicate significant cultural differences between eastern and western Turkey. Since research examining other research samples and methods^22, 23^ has also suggested that participants from eastern Turkey are more holistic than their fellow citizens originating from western Turkey, we considered these two groups as two separate samples that exhibit the above-mentioned expected differences.

## Methods

### Experimental procedure

#### Research ethics

The Research Ethics Committee of Masaryk University reviewed the application to conduct the research project and has approved this project (Proposal No.: 0257/2018) to be conducted on 13 March 2019. The nature of methods used, and their administration were performed in accordance with the relevant guidelines and regulations of Masaryk University and national laws. The necessary approvals and permissions to conduct this research were obtained from participating Universities in Ghana (University of Ghana), Turkey (Adnan Menderes University), and Taiwan (National Chengchi University). All local guidelines were followed during the data collection. Informed consent was obtained in writing from all participants.

Virtual reality *and eye-tracking hardware and software*: HTC Vive Pro head-mounted displays were used with Pupil Labs binocular add-ons to collect eye-tracking data with an estimated accuracy of 0.84° and precision of 0.16°^24^. The sampling frequency of the eye-tracker was set to 90 Hz to correspond to the default refresh rate of the head-mounted display. The experiment (stimuli sequence, presentation timing) was controlled with a set of utility scripts in the Toggle Toolkit^25^.

#### Procedure

**Figure 2:**
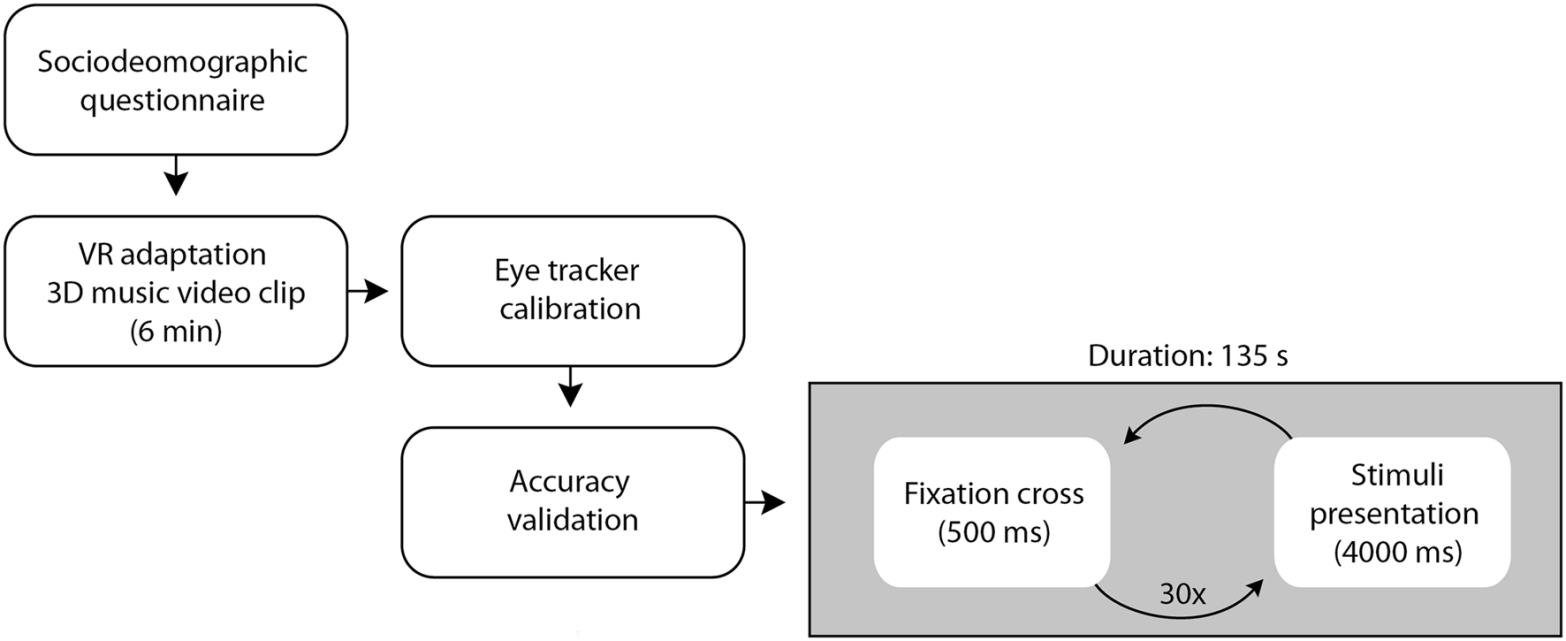
Experimental procedure.

Data gathering was conducted by a trained administrator under the supervision of one of the members of the research team. The experiment was conducted in several steps (Fig. 2), as follows: (1) participants read information about the research and signed the informed consent form; (2) using the Hypothesis software^26^, participants were asked to fill in a sociodemographic questionnaire (size of the municipality participant lives in, gender, age, experience with living in other countries, number of siblings, socioeconomic status, study area, and history of ADHD; for details see Supplement 1); (3) participants underwent a 6-min-long virtual environment adaptation session in a form of stereoscopic video clip to become accustomed to the potentially new experience in virtual reality and to learn the basics of controlling the virtual environment (e.g., moving their heads, an action some participants commonly avoid doing during the first few minutes in the virtual environment; for more information about this topic, see^27^); (4) the experiment’s session in virtual reality. Both the adaptation and experimental sessions were conducted with participants seated, and with no extra movement, constraints were imposed on them.

Each session of the experiment started with the calibration of the eye-tracker using the Pupil Labs native calibration application (6-point calibration with three levels of depth). Although the native calibration application provides feedback to the experiment’s administrator about successful calibration, we added a validation procedure to let us check the accuracy of eye movement during validation. The administrator instructed participants to fixate objects on the validation screen and in the case of insufficient accuracy the eye-tracker was recalibrated. If a particular participant repeatedly failed to pass the validation process, data collection for that participant was discontinued. Furthermore, during the data cleaning stage, we incorporated the validation data to ensure the integrity of our dataset (for more details, see the “Data analysis and cleaning procedure” section). After successful calibration and validation, the participants were presented with 30 trials of static virtual environments. The participants were instructed to “*view the scenes freely*” and informed that “*they will be asked for an evaluation of some of the scenes afterward*”. Before the presentation of each scene (4 s per trial), a fixation cross was displayed for 500 ms. After the presentation of all trials, respondents were asked to a) describe in their own words, and b) evaluate how much they liked (very pretty–very ugly) in a subset of the presented scenes. The responses are not used in this study.

### Materials

To ensure cross-cultural equivalence and to avoid biases on item level caused by inappropriate adaptation^28^, all verbal parts of the experiment (i.e. the questionnaire, the instructions) were back-translated by two independent native speakers^29^ from various cultural backgrounds^30^. The instructions were as simple as possible to prevent or at least minimize any possible administration biases.

The stimulus material consisted of 30 three-dimensional virtual environments (two for training and 28 for testing). Trials were presented in pseudo-random order (a priori randomized and presented in the same order to all participants and groups). The virtual environments were created in Unity Engine using freely available 3D assets^31^. Each virtual environment consisted of two visually and semantically salient focal objects (e.g., cars and car wrecks, barrels, vases, motorcycles) placed in a complex background (typical background components included sky, horizon, vegetation, rocks, and other less salient man-made objects). Both the general virtual environment (background) and the objects were validated in cooperation with the local experts with the aim to minimize possible cultural influences such as familiarity with the scenes and the objects—all parts of the scene had to be commonly present in all cultures included in this research. Such a step of involving scholars from local cultures should increase the construct equivalence^32–34^.

In accordance with Brouwer’s note^13^ that too much detail in stimuli may distract the observers’ attention, we deliberately opted for simpler graphics than the utilized immersive virtual reality technology allows for (e.g., photorealistic digital twins of buildings^35^). Virtual environments also lacked locally specific distinct landscape features and culturally specific objects that could be completely unfamiliar to some cultural groups. On the contrary, the design aimed at commonly used objects.

**Figure 3:**
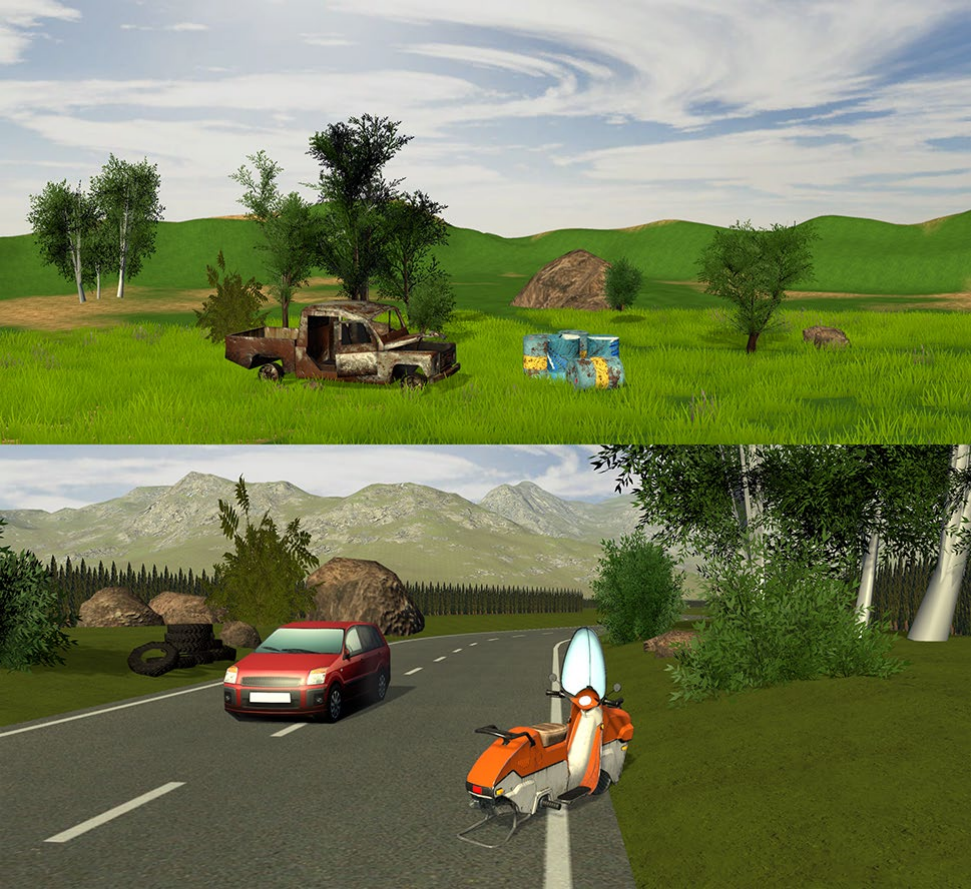
Stimuli—two examples of the presented complex environments. In the upper image the truck wreck and a group of barrels (top) represent focal objects at the same distance from the viewer, whereas in the bottom image, the scooter is nearer to the viewer than the red car.

We used two general virtual environment types in the experiment: (1) scenes in which both focal objects were placed at the same distance from the viewer (Fig. 3 upper part; 16 trials); (2) scenes in which one of the focal objects was placed farther from the viewer (Fig. 3 lower part; 12 trials). Each stimulus was presented for four seconds.

#### Eye-tracking data processing

**Figure 4:**
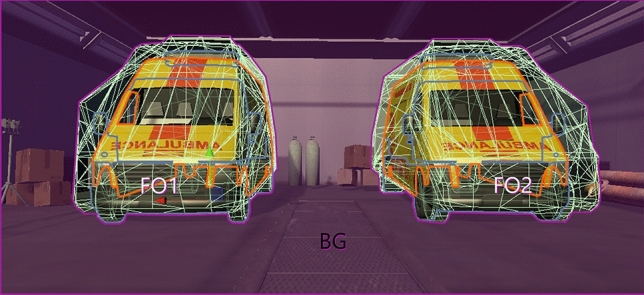
Object colliders. Each focal object (ambulances) possessed an invisible mesh as a collider, set to 110% of the object’s size. These colliders served as regions of interest for the eye tracker. All other objects were marked as background.

Using Unity, we defined object colliders for each focal object since the experiment involved analysis of dwell times on various objects (focal objects and background). These colliders are the 3D equivalents of regions of interest in 2D eye-tracking studies. To correctly classify gaze points, colliders were defined as convex meshes to 110% of the size of the objects (i.e., the colliders did not directly occupy the object’s contours but filled the small space around the objects, Fig. 4). Gaze points on all other objects were coded as background gazes. Other research teams (e.g.,^36^) have previously adopted a similar procedure for studying object hits in virtual reality eye-tracking and provided reliable data while also eliminating the potential inaccuracy encountered with virtual reality eye-trackers.

In the next step, we executed a data cleaning script that filtered corrupt or inaccurate data. Since 50 ms is commonly recognized as a threshold for any cognitive recognition^37^, we considered gazing at an object for more than 50 ms a visit and rejected gazes below this limit. If a visit was followed by a very short hit (less than 32 ms) on another object followed by a return to the original object, the duration of the gaze on this object before and after the interruption was merged into one visit to prevent data distortion caused by measurement inaccuracy. Dwell times were calculated for each object category (focal object 1, focal object 2, background) as the sum of the duration of all visits. Data loss (blinks, closed eyelids, eye-tracking desynchronization, etc.) was computed per stimulus in addition to focal object and background dwell time.

#### Data analysis and cleaning procedure

Before data collection, we conducted a power analysis for one-way ANOVA in G*Power (v3.1.9.7^38^) with the following settings: *f* = 0.25, α = 0.05, 1 − β = 0.80, number of groups = 5. The effect size was determined according to previous research by Duan^10^, who reported large effect sizes for eye-movement data (η_p_^2^ ranging between 0.12 and 0.77). We decided on a relatively more conservative strategy at the medium effect size threshold. The results specified a minimum sample size of 200 participants (40 per group) to achieve 80% statistical power.

As mentioned above, we gathered virtual reality data from five cultural samples: Czechia, Ghana, Taiwan, eastern Turkey, and western Turkey. All participants were students enrolled at local universities. The participation was advertised in university courses and via student unions. The only exclusion criterion was the presence of severe uncorrected eye defects. Considering the potential experimental mortality typical of this kind of research (e.g., poor data quality and data loss, not all participants completing the experiment), we obtained 297 participants in total. The data were collected as part of a larger test battery that investigated cross-cultural differences in visual perception and included other tasks such as Navon Figures based methods^7^, and complex 2D scenes^6^. Results of those methods have not been published yet. For various reasons, some participants from each country could not be measured or included in the analysis, mainly because of impracticable pupil detection or nausea during the experiment. In this stage, 10 participants were excluded because of technical issues in the data collection process.

To ensure good data quality, we performed a data cleaning procedure. In the first step, we calculated the average data loss from the data loss per stimulus (% of missing eye-tracking records). The threshold for removing a participant was set arbitrarily at 5% of missing records. Twelve participants were removed because of high data loss. The average data loss in the remainder of the sample was 0.6%. The second step involved a qualitative assessment of the validation procedure by two independent evaluators (researchers with high expertise in eye-tracking technology) who rated the quality of each individual validation. The records from the validation scene of each participant were exported by a technical worker and subjected to visual inspection with an emphasis on the accuracy of gaze points on target objects and the absence of undesirable artifacts. Only in the case of agreement on the sufficient quality of the recording were the participants' data accepted for analysis. An additional 21 cases were removed based on poor validation. In the final step, we removed 12 participants based on their reported history of ADHD. The entire cleaning procedure resulted in a dataset of 242 participants (for more details see the next section).

To test the first hypothesis, we performed a one-way ANOVA. First, we summed the duration of visits on both focal objects to obtain a single aggregate focal object dwell time per stimulus; we then calculated the mean focal object dwell time across all 28 test trials. Then, we verified the ANOVA assumptions. Because Levene’s test indicated unequal variances across groups (*F* = 5.44, *p* < 0.001) and the Shapiro–Wilk test suggested normally distributed residuals (*W* = 0.99, *p* = 0.65), the Welch’s degrees of freedom correction for one-way ANOVA was conducted. We also applied Games–Howell post-hoc tests with Tukey HSD correction.

To test the second hypothesis, we performed a mixed ANOVA. Only the twelve stimuli with focal objects at varying distances from the viewer were subjected to this analysis. We calculated the mean dwell time for both types of focal objects. Regarding ANOVA assumptions, the data indicated unequal variance across groups (*F* = 6.82, *p* < 0.001), sufficient sphericity (*p* > 0.999), and non-normal distribution of residuals (*W* = 0.99, *p* < 0.001). Three cases (one from Ghana, one from eastern Turkey, and one from western Turkey) were identified as outliers based on a visual inspection of the quantile–quantile plots and consequently removed from the analysis. A repeated check of the ANOVA assumptions suggested unequal variance across groups (*F* = 6.86, *p* < 0.001), spherical data (*p* > 0.999), and normal distribution of residuals (*W* = 9.99, *p* = 0.111). Since Welch's correction is not implemented for mixed ANOVA and at the same time ANOVA is relatively robust for violation of equality of variances, we conducted mixed ANOVA with focal object dwell time as a dependent variable, cultural group as a between-subject factor and type of object (type: closer, further) as a within-subject factor. To overcome issues with inequality of variance, omega squared effect size is reported. All *p*-values were adjusted using the Holm–Bonferroni method.

We analyzed the data in R using the following packages: *afex*, *effsize*^39^, *ggforce*, *ggdist*^40^, *gghalves*, *lme4*, *lsr*, *performance*^41^, *psych*^42^, *rstatix*, *rmisc*, and *tidyverse*. The data matrices and *R* code containing the data cleaning procedure are available online at the URL https://osf.io/2kqts/.

#### Research sample

The entire data cleaning procedure resulted in a dataset composed of 242 participants representing five cultural samples: Czechia (42), Ghana (39), Taiwan (51), Turkey East (56), and Turkey West (54). See Table 1 for per-country descriptives of gender and age. For detailed sample descriptives (e.g., socio-economic status, number of siblings, size of municipality of birth, study area), see Supplement 1.

**Table 1:** Sample descriptives *(N* = *242*).

Country	% of females	Age: mean (SD)	Age: range
Czechia (N = 42)	66.7	22.3 (2.08)	18–26
Ghana (N = 39)	46.2	21.3 (0.95)	20–24
Taiwan (N = 51)	68.6	21.8 (2.09)	20–29
Turkey East (N = 56)	33.9	22.4 (1.92)	19–30
Turkey West (N = 54)	25.9	22.0 (1.88)	19–27

## Results

### Overall average dwell on focal objects

**Figure 5:**
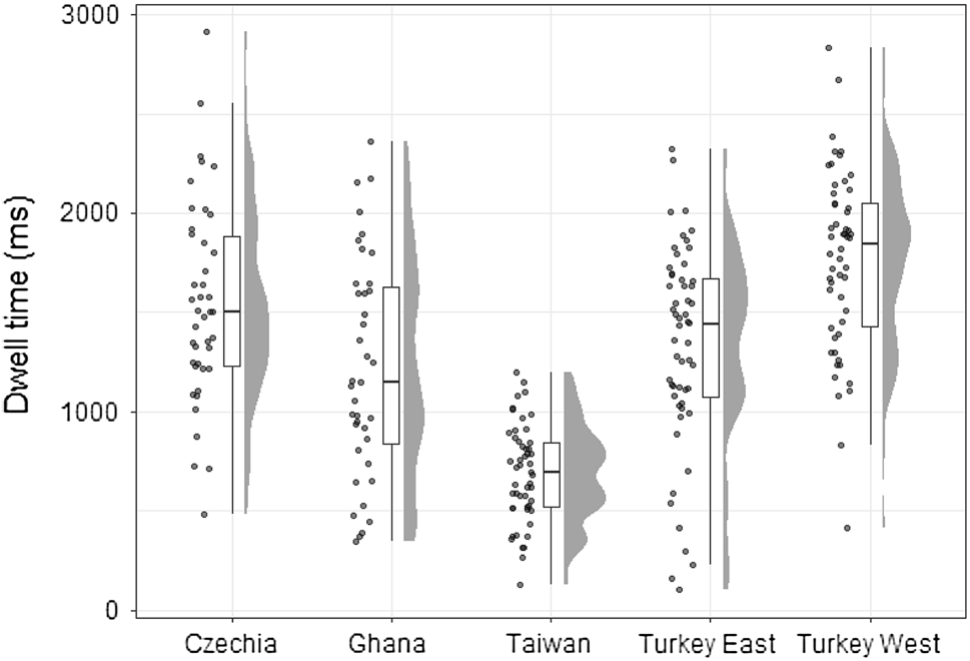
Focal object dwell time per country.

In the eye-movement analysis, we focused on visits and the respective dwell time on focal objects. Figure 5 shows per-country distributions of the mean focal object dwell time. The means and confidence intervals for each country are summarized in Table 2.

**Table 2:** Focal object dwell time descriptives per country (in milliseconds; N = 242).

Country	Mean (*SD*)	lower 95% *CI*	upper 95% *CI*
Czechia	1547.14 (506.07)	1389.44	1704.85
Ghana	1217.24 (554.06)	1037.635	1396.85
Taiwan	687.81 (244.53)	619.04	756.59
Turkey East	1326.89 (511.99)	1189.78	1464.00
Turkey West	1760.77 (458.79)	1635.54	1885.99
Total sample	1309.58 (590.94)	1234.75	1384.41

Next, we tested for differences between cultural groups. ANOVA revealed a statistically significant effect of the cultural group on the mean of focal object dwell time with a large effect size, *F*(4, 108.84) = 73.93, *p* < 0.001, ω^2^ = 0.386. Post hoc tests (Table 3) indicated that the Taiwanese spent significantly less time observing focal objects than participants from all the other countries. Participants from western Turkey spent significantly more time observing focal objects than all the reported groups apart from Czechia. No significant difference was observed between participants from eastern Turkey and Czechia or Ghana. Participants from Czechia spent significantly more time observing focal objects than participants from Ghana. All significant pairwise differences showed large effect sizes and insignificant comparisons indicated weak to medium effect sizes. Thus, this hypothesis was partially supported. See the Supplement for comparison of dwell time on focal objects and background.

**Table 3:** Dwell time post-hoc tests: p-values and Hedges g [95% CI].

Comparison	Mean difference [95% *CI*]	*p*-value	Hedges *g* [95% *CI*]
Czechia—Ghana	330 [47, 613]	0.050	0.62 [0.17, 1.06]
Czechia—Taiwan	859 [594, 1124]	< 0.001	2.21 [1.69, 2.73]
Czechia—Turkey East	220 [− 39, 480]	0.220	0.43 [0.03, 0.83]
Czechia—Turkey West	− 214 [− 475, 48]	0.215	− 0.44 [− 0.85, − 0.04]
Ghana—Taiwan	529 [259, 800]	< 0.001	1.29 [0.83, 1.74]
Ghana—Turkey East	− 110 [− 375, 156]	0.864	− 0.21 [− 0.61, 0.20]
Ghana—Turkey West	− 544 [− 811, − 277]	< 0.001	− 1.08 [− 1.51, − 0.64]
Taiwan—Turkey East	− 639 [− 885, − 393]	< 0.001	− 1.56 [− 1.99, − 1.12]
Taiwan—Turkey West	− 1072 [− 1322, − 825]	< 0.001	− 2.87 [− 3.42, − 2.32]
Turkey East—Turkey West	− 434 [− 676, − 191]	< 0.001	− 0.89 [− 1.27, − 0.49]

### Dwell time on closer and further objects

**Figure 6:**
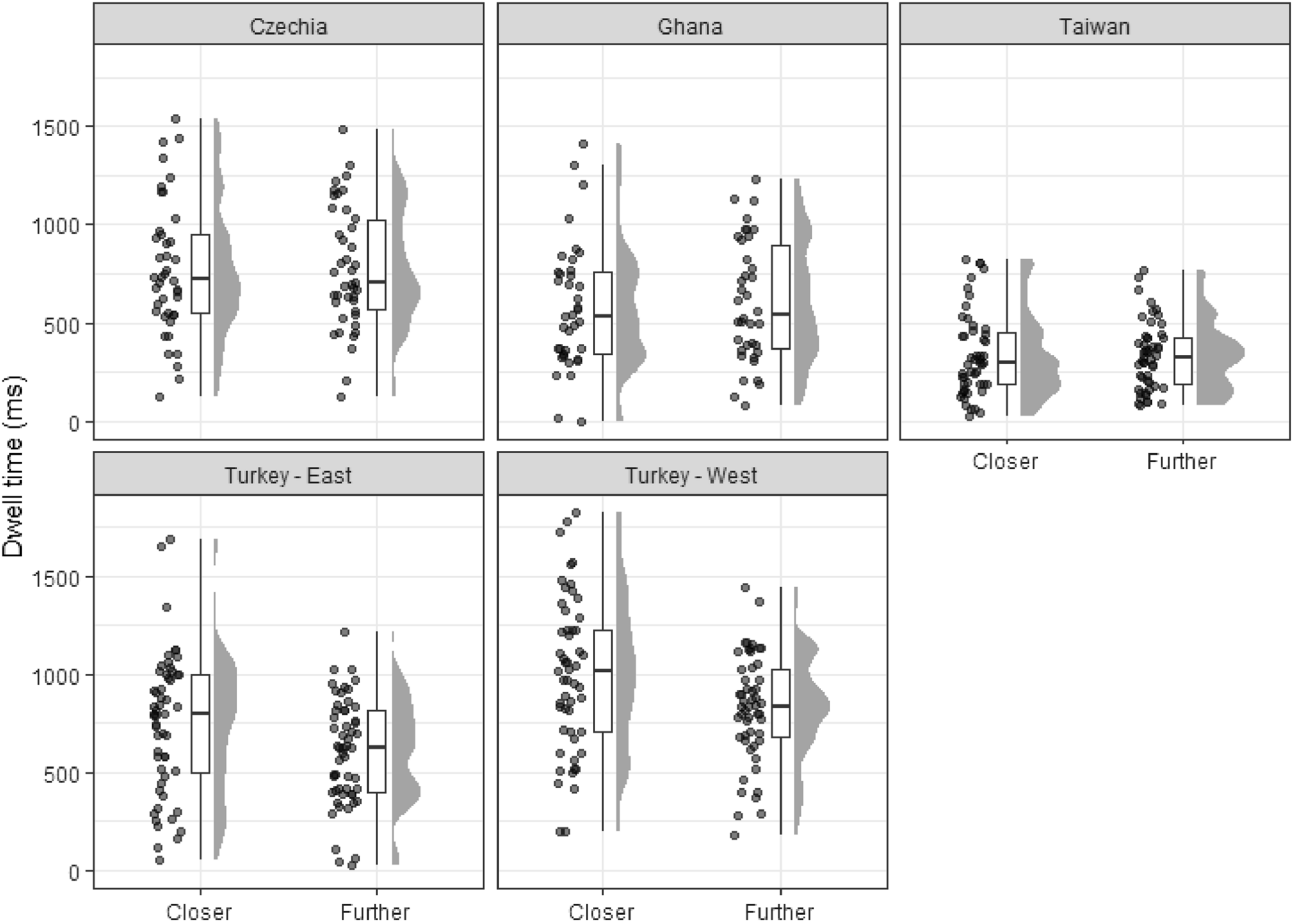
Dwell time on focal objects closer and further from the viewer.

In the next step, we examined the potential role of depth in relative focus on focal objects placed closer or further away from the viewer. See Fig. 6 and Table 4 for details.

**Table 4:** Type of focal object dwell time descriptives per country (in milliseconds; N = 239).

Country	Type of FO	Mean (*SD*)	lower 95% *CI*	upper 95% *CI*
Czechia	Closer	770.43 (339.08)	664.76	876.09
N = 42	Further	775.99 (310.30)	679.29	872.68
Ghana	Closer	581.55 (320.59)	476.18	686.93
N = 38	Further	608.56 (310.45)	506.51	710.60
Taiwan	Closer	336.87 (215.44)	276.27	397.46
N = 51	Further	331.73 (172.45)	283.22	380.23
Turkey East	Closer	746.11 (357.93)	649.34	842.87
N = 55	Further	601.53 (268.25)	529.01	674.05
Turkey West	Closer	998.57 (391.08)	890.77	1106.36
N = 53	Further	824.87 (271.19)	750.11	899.62
Total sample	Closer	692.88 (399.61)	641.96	743.80
	Further	625.26 (318.96)	584.62	665.90

We performed a mixed ANOVA. The main effect of the country was statistically significant, with a large effect size *F*(4, 234) = 34.72, *p* < 0.001, ω^2^ = 0.103, therefore supporting the results of the previous one-way ANOVA. The main effect of the type of object (i.e., object distance from the viewer) was also statistically significant, with a medium effect size *F*(1, 234) = 8.84, *p* < 0.001, ω^2^ = 0.008, suggesting that if we investigate all the participants regardless of their country, on average they spend more time on objects which are closer than further. Finally, the interaction between cultural group and object type was also significant, with medium effect size *F*(4, 234) = 4.71, *p* < 0.001, ω^2^ = 0.009.

Since the interaction was significant, a more detailed analysis of the results was conducted via simple main effects. The simple main effect of cultural group was significant for both closer, *F*(4, 234) = 28.00, p < 0.001, η^2^_g_ = 0.324, and further objects *F*(4, 234) = 26.30, p < 0.001, η^2^_g_ = 0.310. For these differences, however, we did not state any hypotheses, and therefore we did not perform additional post-hoc tests. The simple main effect of the focal object position was significant for Turkey East and Turkey West participants, with medium to large effect sizes. In both of these cultural groups, the participants gazed at the closer focal objects for a longer time. No significant difference was evident in the other three cultural groups, with negligible effect sizes (see Table 5 for details). Since the type of focal object was a binary variable, no post-hoc tests were performed. Overall, the hypothesis concerning systematic cross-cultural differences in depth perception was not fully supported since they were observed only among participants from both Turkish groups.

**Table 5:** Per country focal object type dwell time post-hoc tests.

Country	Mean difference closer-further (ms)	F	*p*-value	η^2^_g_
Czechia (N = 42)	− 5.56 [− 158.10; 146.95]	0.02	1.00	< 0.001
Ghana (N = 38)	− 27.00 [− 187.33, 133.3]	0.40	1.00	0.002
Taiwan (N = 51)	5.14 [− 133.25, 143.54]	0.02	1.00	< 0.001
Turkey East (N = 55)	144.58 [11.31, 277.84]	11.4	0.005	0.051
Turkey West (N = 53)	173.70 [37.94, 309.46]	11.8	0.005	0.064

### Dwell on objects and other individual-level variables

In the last step, we performed an exploratory analysis of possible influence of other variables on the dwell time on focal objects. We performed ANCOVA with dwell time on focal objects as the dependent variable, culture as the independent variable, and size of the municipality the participant lives in, gender, age, experience with living in another country, number of siblings, socioeconomic status, and study area as covariates. Apart from a small to moderate effect of gender (*p* = 0.010, η_p_^2^ = 0.051), no other variable was in a significant relationship with the dependent variable. The main effect of gender is associated with a decrease in the average dwell for females compared to males. The model with covariates showed the same pattern of differences in focal object dwell time across countries as the basic model (for full results, comparison of models and descriptives by country and gender, see Supplement 1).

## Discussion

The paper presents the results we obtained from a study of the cognitive processing of complex visual stimuli in a sample of five cultural groups native to Czechia, Ghana, Taiwan, eastern Turkey, and western Turkey. We used immersive virtual environments as stimuli and an eye-tracking device to measure the participants’ oculomotor behaviors to assess the distribution of attention during a presentation of several virtual environments. Recent similar studies using 2D stimuli were inconclusive on the cross-cultural variance in focusing on salient objects (analytic cognitive style) or on the scene in its entire complexity (holistic cognitive style)^6, 7^, as originally reported by Masuda and Nisbett^8^.

2D images have been previously questioned as suitable proxies for real objects^43^), whereas 3D representations in an immersive virtual environment appear to provide a potential alternative for further research. Sharing the optimism of de la Rosa and Breidt^44^ on the potential of using virtual reality to enhance the quality of psychological research, we transferred two-dimensional scenes into a three-dimensional virtual world to provide greater ecological validity^45, 46^ and the option to include the aspect of depth make virtual reality a suitable tool for examining the aforementioned theories of cross-cultural differences.

In the first presented analysis, which examined the distribution of attention between visually salient focal objects and complex background, the results partially supported the geographic distribution of the cognitive styles we expected according to the assumptions by Masuda and Nisbett^8^. Taiwanese participants spent significantly less time on focal objects than on the rest of the scene, an observation that supports their position as typical representatives of holistic cognitive style. This difference was convincing, especially compared to participants from Czechia, or western Turkey. Interestingly, however, western Turkey was distinct, proving to be the most analytic sample.

We achieved the aim of capturing the possible cultural differences within the Turkish sample: the observed differences in oculomotor behaviour between both Turkish samples agreed with the previously detected within-country differences in cognitive style^4, 23, 47^. These differences might be explained by the above-mentioned present-day socio-demographic differences in Turkey and other historical factors. Asia Minor has been a border between Mediterranean and Eastern influences for centuries, and although the western area was under the continuous influence of the Byzantine Empire until 1453, the eastern area experienced significant influence from Persian, Arab, and Seljuk expansion. Differences can also be observed in the second half of the twentieth century, when a large portion of the workforce heading for West Germany was recruited from West Anatolia, Istanbul, and Ankara^48^. A large portion of these workers and their second-generation offspring remigrated back to their homeland which might have reciprocally influenced their home culture.

Surprisingly, the Czech Republic, which was hypothesized as the most analytical, was not distinct from the other studied samples. The object/background attentional patterns for the Czech sample were very similar to the patterns of the sample from Turkey. Whether forced collectivization or other historical phenomena may have caused this variance in central and western European countries in cognitive processes as suggested by Varnum^49^, the underlying perception and evaluation of complex stimuli remains a question. The present study, however, reports some of the first research in the field, comparing the oculomotor patterns of participants beyond commonly studied samples using diverse samples from central Europe, western and eastern Asia, and Africa, and applying methods in a 3D virtual environment^50^. It supports the original theory stating that cultural background affects visual perception, and that culture may be understood as a condensed experience that determines individuals’ perceptual schema^51, 52^ that drives the exploration activity in the top-down direction^53^. In the second analysis concerning the differences in processing scenes with focal objects at varying distances, both Turkish samples spent relatively more time looking at closer focal objects. No statistically significant difference of this kind was observed in samples from Czechia, Ghana, and Taiwan.

In summary, we can draw three main conclusions from the present study and the use of virtual reality combined with eye-tracking. A person’s cultural group plays a role in visual attention distribution in virtual environments, resp. in their relative attention to focal objects. Second, the findings of significant differences within both Turkey samples underscore the importance of not thinking about countries as culturally monolithic phenomena but being aware that aggregation of data at the national level may camouflage meaningful differences at the within-culture level (see^54^).

Finally, third, there are notable differences in perception of depth (and respectively relative attention to closer and further objects) across the countries, but they are not systematic and easily interpretable at this point. Whereas in Taiwan, Czechia, and Ghana there were practically no differences in attention to closer and further objects detected (differences between 5 to 25 ms), in the case of both Turkish samples they were distinct (145, resp. 174 ms). We find this difference in the patterns of observation of closer and further objects quite interesting, moreover, both cohorts significantly differ in the overall attention to focal object vs. background.

Our research is not without limitations. Generalization of the results is limited by the novelty of the technology: to eliminate the differences in digital literacy of the participants in the study, we selected a student population in which we expected less pronounced differences in digital skills. This particular generation is less likely to have technology-related biases, however, at the same time is most globalized and probably demonstrates a lower level of the culturally based differences the study was aiming at.

The scenes, used as visual stimuli, contain a variety of smaller and larger objects, making them complex in terms of content. However, it is important to distinguish between the complexity of the scene's content and the photorealistic quality of its rendering. In our study, we focused on the former, emphasizing the diversity and arrangement of visual elements rather than striving for photorealism. Manipulation with the level of photorealism of 3D environments^55–57^ and observation of the effects of this manipulation on the participants' perception and its cross-cultural specifics could offer intriguing prospects for further research.

Apart from this, further cross-cultural eye-tracking research should focus on more controlled manipulations with the objects within the virtual visual field related to monocular and binocular depth cues^58^. For example, on perception and interpretation of conflicting cues, changes in spatial position, and perception of moving objects.

Finally, an important limitation of this study concerns the operationalization of culture within our cross-cultural research framework. We acknowledge that the issue of equating location with culture is a persistent challenge in quasi-experimental laboratory studies of this nature. While exploring more refined cultural subgroups or local cultures within each studied region might offer valuable insights, it would necessitate larger samples and the inclusion of established culture measurement scales, such as individualism/collectivism or independent/interdependent self-construal, as explanatory variables. However, our previous attempts at validating these methods encountered difficulties^28, 59^, and we found it necessary to prioritize further developments in culture operationalization methods for more accurate cross-cultural comparisons.

### Supplementary Information

Supplementary Information.

## Data Availability

The dataset, data-analytic scripts (in R), and supplementary material are accessible in the Open Science Foundation (OSF) repository at the following URL: https://osf.io/2kqts/. This study was not preregistered.
